# An Underrated Diagnosis of Superior Mesenteric Artery Syndrome: A Case Report

**DOI:** 10.3390/diagnostics12092159

**Published:** 2022-09-06

**Authors:** Irina Ciortescu, Roxana Nemteanu, Corina Hincu, Liliana Gheorghe, Alina Plesa

**Affiliations:** 1 Medical I Department, “Grigore T. Popa” University of Medicine and Pharmacy, 700115 Iași, Romania; 2 Institute of Gastroenterology and Hepatology, “Saint. Spiridon” Hospital, 700111 Iași, Romania; 3 Department of Radiology, “Saint. Spiridon” Hospital, 700111 Iasi, Romania

**Keywords:** superior mesenteric artery syndrome, Wilkie’s syndrome, intestinal obstruction

## Abstract

Superior mesenteric artery syndrome (Wilkie’s syndrome) is a rare cause of intestinal obstruction caused by a congenital or acquired reduction of the aorto-mesenteric angle leading to duodenal compression. We present the case of a 51-year-old female patient with a previous history of breast cancer. She was admitted to the Emergency Department with acute onset of recurrent vomiting, intense abdominal pain especially in the epigastric region, and abdominal distension. The ultrasound showed an absence of lower abdominal quadrants with an enlarged and distended stomach reaching the pelvis. The computer tomography scans confirmed the diagnosis of superior mesenteric artery syndrome. Conservative management was implemented, and using a nasogastric tube, and upped endoscopy approximately 4000 mL of fluid were aspirated with clinical improvement shortly after. The patient resumed a high-calorie diet and five months later, the patient was completely asymptomatic.

Superior mesenteric artery syndrome (SMAS) is a rare gastrointestinal (GI) and vascular medical condition, with a prevalence of less than 0.5% in the general population [1]. The symptoms associated with SMAS such as epigastric pain, early satiety, abdominal distension, and vomiting frequently overlap other, more common diseases and may lead to diagnostic delay [2]. Historically, Carl von Rokitansky, followed by David Wilkie, was the first who identified, thoroughly described and later on named this disease in 1927 (Wilkie’s syndrome) [3,4]. The majority of SMAS cases are acquired syndromes, involving mostly women who lost weight voluntarily or secondary to other medical conditions, such as malignancy, eating disorders, anorexia, malabsorption, or gastrectomy. and, as such, lost the perivascular mesenteric fat that kept a wide aorto-mesenteric angle [2,5]. The reduction of fat reduces the angle between the vessels, the duodenum, and the left renal vein, and consequently, patients become symptomatic. The congenital form of SMAS is rare, but usually easier to diagnose because patients develop symptoms from early childhood [2]. Patients with mild obstruction may have epigastric pain, gastroesophageal reflux, and early satiety, whilst those having significant obstruction usually describe emesis, weight loss, and recurrent bilious vomiting. The initial non-surgical treatment implies enteral and parenteral nutrition with a high-calorie diet intake which is sufficient to restore the perivascular fat and permeability. Insertion of a jejunal feeding tube may be required, but the majority of patients recover with an oral diet [6]. Elderly patients with SMAS are often considered unsuitable candidates for surgical decompression due to advanced chronic, long-standing, or terminal illnesses which are responsible for vascular compression syndrome [7]. SMAS may be associated with left vein stenosis, and when symptomatic (microhematuria, varicocele, left flank pain, and vascular thrombosis), the phenomenon is called the Nutcracker syndrome [8]. The co-existence with SMAS is common, and the left renal vein may run anterior to the aorta or posterior to the aorta, retro-aortic, compressed between the spine and the abdominal aorta. The initial treatment for both medical conditions implies a non-surgical approach using enteral and/or parenteral nutrition with a high-calorie diet intake which is sufficient to restore the perivascular fat and permeability. Insertion of a jejunal feeding tube may be required, but the majority of patients recover with an oral diet. However, in some cases, surgery (lateral-lateral duodenojejunostomy or Roux-en-Y reconstruction) or endovascular stent placement (in case of significant left renal vein stenosis) may be recommended [9]. In addition, infrarenal transposition of the SMA into the infrarenal aorta or endoscopic ultrasound-guided gastrojejunostomy may be useful in selective cases [10,11]. The goal of a successful treatment is weight gain and optimal relief of digestive symptoms which are obtained after the adequate restoration of perivascular fat tissue and normal angulation of the aorto-mesenteric angle.

We present the case of a 51-year-old female patient admitted to the Emergency Department with acute onset of recurrent vomiting, intense abdominal pain especially in the epigastric region, and abdominal distension. The patient declares a 3-months history of early satiety, low appetite, and intermittently mild postprandial abdominal pain, without any significant weight loss. Her background included a prior history of breast cancer with complete mastectomy and lymphadenectomy with a favorable long-term prognosis. Clinical examination revealed a slender patient with a body mass index (BMI) of 18.1 kg/m^2^ and a distended, diffusely painful abdomen (Figure 1). Her weight has fluctuated lately with a decrease in appetite, but she does not report significant weight loss. However, during the last few months, she claimed to have early satiety, and intermittently mild postprandial abdominal pain, and she was prescribed proton pump inhibitors and prokinetic medication to manage her symptoms. Her laboratory parameters were unremarkable and showed mild leukocytosis and iron deficiency anemia with a hemoglobin level of 10.8 g/dL, a mean corpuscular volume of 78 fL, serum iron level of 35 µcg/dL, and ferritin level of 12 ng/mL, without evidence of a GI bleed. The urinary analysis report showed microhematuria. Her kidney function was within the normal range, and the urinary culture was negative.

Immediate exploration of the abdomen was performed in the Emergency Department using X-rays, ultrasound, and a computed tomography (CT) scan of the abdomen. The abdominal ultrasound revealed an enlarged stomach (Figure 2), and the CT showed a vascular abnormality with a reduction of the aorto-mesenteric space of 21.6° and a distance (3.54 mm) obstructing the third portion of the duodenum, and partial left renal vein stenosis confirming the diagnosis of SMAS and Nutcracker syndrome (Figure 3).

The abdominal X-ray showed significant gastric and duodenal distension and a nasogastric (NG) tube was inserted to prevent aspiration. A total of 4000 mL of fluid were drained after insertion of the NG tube. An upper endoscopy was performed and showed no associated or obscure lesions in the upper GI tract (Figure 4). The surgical team opted for conservative management and fluid replacement therapy in association with NG feeding was initiated. The patient started an initial enteral and parenteral enriched fluid diet. By day 5, the patient showed no symptoms and was able to tolerate an oral diet of 2000 kcal per day. She was discharged and a high-calorie diet intake was recommended. By her fifth month follow-up appointment, the patient was completely asymptomatic, with a BMI of 20.2 kg/m^2^.

SMAS is a rare entity and can affect both genders and all age groups [12]. Even though there are numerous publications, case reports, and single-center reports in the literature regarding this syndrome, the awareness among clinicians is still very low [11,12]. The reported overall incidence is low up to 0.3% but is higher among burn victims, trauma, brain injury, and patients undergoing corrective spinal surgery for scoliosis due to vertical traction, with an incidence rate up to 2.4% [13]. Corrective surgery required for spinal injury or curvature treatment enable the appearance of SMAS because it elongates the spine cranially, shifts the origin of SMA, and reduces the aortomesenteric angle [13].

As previously mentioned, the third portion of the duodenum is positioned at the angle formed by the aorta and the superior mesenteric artery. A narrow-angle would enable the obstruction of the third segment by the arterial vessels due to a loss of retroperitoneal and mesenteric fat deposits which are surrounding the vascular structures [4,14]. The normal angle between the aorta and the superior mesenteric artery (measured on the mid-sagittal image between the abdominal aorta and mesenteric artery) is considered to be from 25–65° [15]. In patients with SMAS, the angle is reduced below 25° [16]. Equally important is the aorto-mesenteric distance (measured on the axial image at the level of the horizontal third part of the duodenum between the abdominal aorta and mesenteric artery) which is also reduced from a normal range of 10–28 mm to below 10 mm in SMAS [15]. Furthermore, left renal vein compression may also be present and patients may be completely asymptomatic or might develop symptoms such as hematuria, varicocele, pain in the left flank, or even renal vein thrombosis, a syndrome called Nutcracker syndrome [17]. The association of both syndromes is a common occurrence. Nutcracker syndrome, also known as left renal vein entrapment, is characterized by an impaired venous outflow from the left renal vein into the inferior vena cava due to extrinsic compression, often accompanied by obvious dilatation and narrowing [8,17]. The lack of retroperitoneal fat which can reduce the aortomesenteric angle, causing vascular compression, is the main culprit. The syndrome may imply compression of the vein between the aorta and the SMA, or in other cases the retro aortic renal vein may be compressed between the aorta and the vertebral body, causing the posterior nutcracker syndrome [8]. Hematuria and pain are the most common symptoms described by patients. Hematuria is attributed to the rupture of small vessels due to elevated venous pressure, in the collecting system [15,17]. It varies from microhematuria to macrohematuria and can be associated with anemia. Flank or posteromedial thigh pain may be triggered when walking, sitting, or standing explained by the passing of blood clots [17].

The loss of body fat is associated with a higher risk of developing SMAS [10]. That is why patients with certain illnesses such as psychiatric conditions that are associated with anorexia or vomiting, malignancy, or malabsorption conditions are at risk for developing SMAS. However, not all patients exerting these common conditions associated with weight loss develop SMA which means that other mechanisms may be incriminated. Congenital anatomical changes like a low SMA insertion or a high insertion of the Treitz ligament might be involved [6,7]. The diagnosis is easily misleading mimicking and overlapping other more frequent illnesses. Unfortunately, the BMI score does not reflect the visceral fat for patients with a suspicion of SMAS [11,12]. A CT or a magnetic resonance imaging examination would offer more accurate information regarding the fat body distribution.

Currently, the exact prevalence of vascular compression syndromes is unknown but is generally considered very rare, still poorly understood, and difficult to diagnose. Noninvasive tests such as standard ultrasound examination of the duodenum are insufficient for a positive diagnosis, but Doppler ultrasound offers detailed information concerning the venous and arterial flow, aorto-mesenteric angle, and distance enabling a quick and accurate diagnosis. Ultrasound is a sensitive method, and it has been shown to have a good correlation with CT [13].

Our patient had a history of malignancy with unremarkable but progressive weight loss and mild digestive symptoms before the diagnosis. A correct and early diagnosis would have been difficult to state given the patient’s discrete and unspecific symptomatology which could easily overlap other more common medical conditions. The patient had signs of partial intestinal obstruction such as intermittent nonvolitional vomiting, postprandial epigastric pain, and early satiety shortly after food intake which was confused with other anatomical or motility-related disorders. The acute onset of epigastric pain, plenitude, vomiting, and abdominal distension enabled physicians to adequately explore the digestive tract and diagnose intestinal obstruction secondary to SMAS. Additionally, the patient presented with asymptomatic mild hematuria which was a valuable argument for the diagnosis of left vein compression, in the absence of other causes such as masses, infection, and kidney stones.

The majority of the cases with this vascular-anatomic syndrome are treated conservatively by medical management (decompression of the stomach with an NG tube, correction of nutritional and electrolytes deficiencies, provisionally parenteral nutrition) [2,10]. This approach is advocated for patients who have a short clinical history, similar to the case presented. Our patient was encouraged to regain the lost body fat by correcting calorie intake, and the symptoms regressed as the weight improved. No surgical intervention was needed. At the follow-up, she reported a subjectively normal quality of life. There is no standardized timeline for assessing feedback to conservative management, but usually, an optimal response is observed in a few days. Appropriate dietary adjustments could prevent any future relapse. However, in situations where medical measures fail to improve the patient’s symptomatology, surgical management may be necessary requiring a complicated gastrojejunostomy, duodenojejunostomy, or a Ligament of Treitz release procedure [2].

Vascular compression syndromes are rare medical conditions. However, a clinical diagnosis requires a high index of suspicion and should be kept in mind in cases where patients have recurrent symptoms of vomiting, epigastric pain, postprandial abdominal distension, and recent history of weight loss. The diagnosis is confirmed by a CT scan. Unfortunately, there are no standardized treatment protocols, but conservative medical treatment is the first-line choice. Further studies are necessary to identify characteristics of patients with SMAS (additional risk factors that contribute to disease occurrence and severity) that fail conservative management and undergo complex surgical procedures. Based on our experience, we feel that it is important for physicians to remain alert to the possible association between SMAS and malignancy. Early recognition may allow appropriate management by interrupting the cycle of weight loss and secondary upper gut obstruction from SMAS.

## Figures and Tables

**Figure 1:**
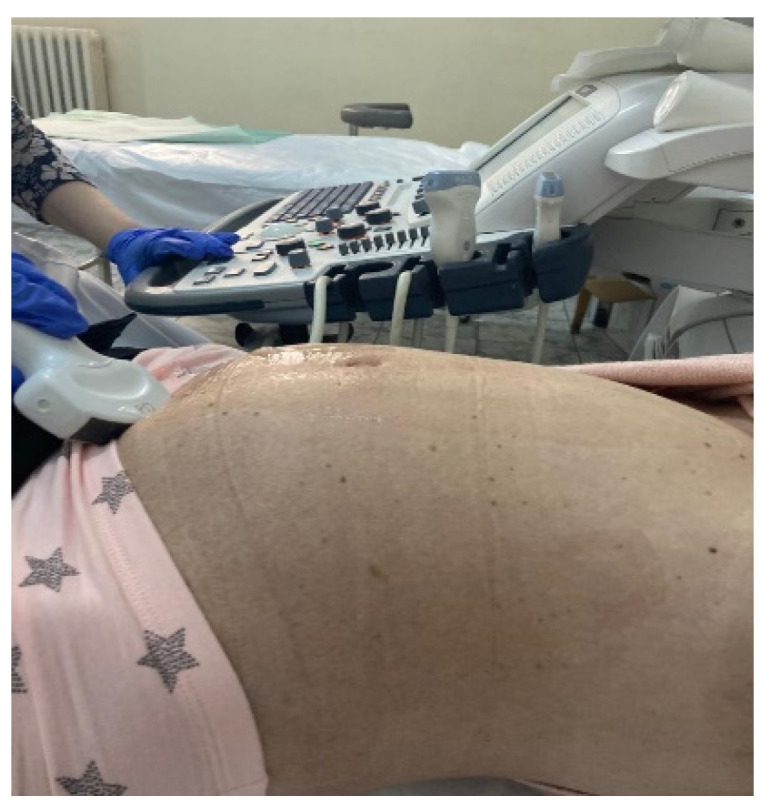
Abdominal distension.

**Figure 2:**
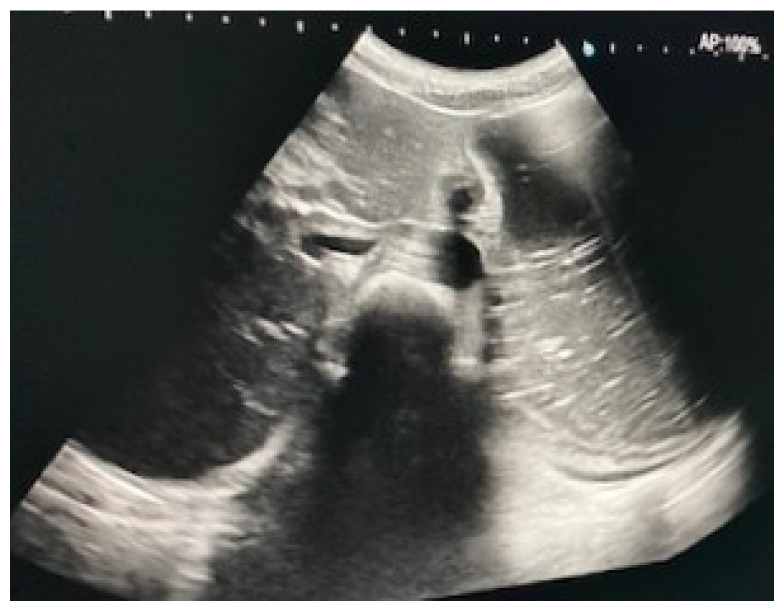
Abdominal ultrasound showing gastric distension.

**Figure 3:**
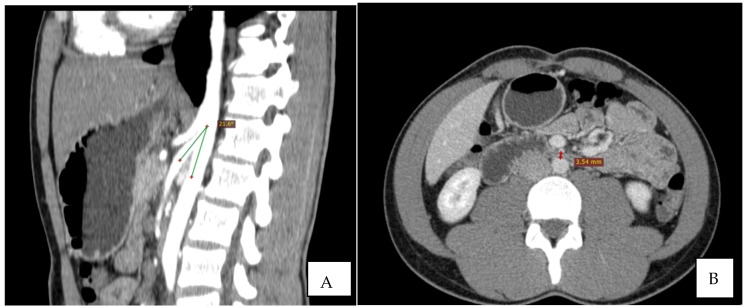
CT: the sagittal reconstruction shows the reduction of aorto-mesenteric angle. ((**A**) beak signand distance (**B**)).

**Figure 4:**
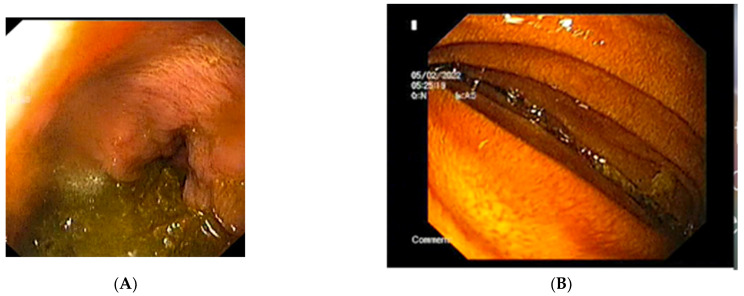
The upper endoscopy shows: (**A**) the stomach containing and (**B**) the extrinsic compression of the third segment of the duodenum.

## Data Availability

Not applicable.
